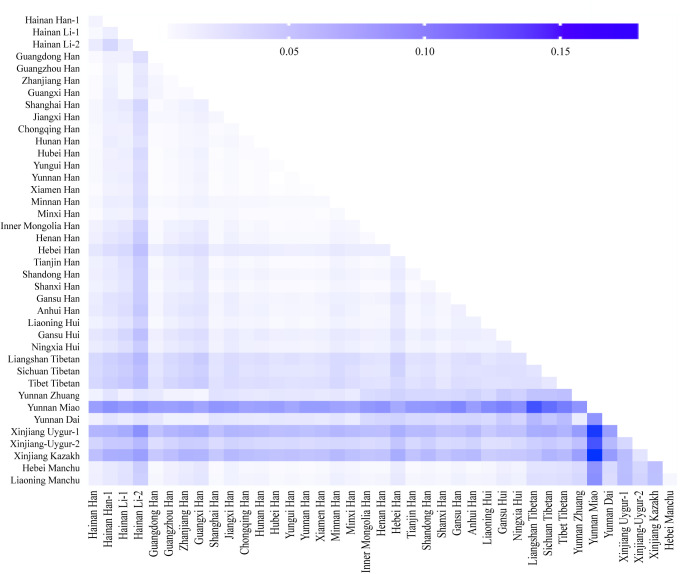# Genetic variation and phylogenetic analysis of 23 STR in Chinese Han population from Hainan, Southern China

**DOI:** 10.1097/MD.0000000000038428

**Published:** 2024-05-31

**Authors:** Xing Zou, Qianyun Nie, Wenhui Li, Yinyu Chen, Tao Song, Peng Zhang

**Affiliations:** a Department of Forensic Medicine, Hainan Provincial Academician Workstation (Tropical Forensic Medicine), Hainan Province Tropical Forensic Engineering Research Center, Hainan Medical University, Haikou, China; b Department of Pathology, School of Basic Medicine and Life Sciences, Hainan Medical University, Haikou, China; c Department of Pathology, The First Affiliated Hospital of Hainan Medical University, Haikou, China.

**Keywords:** forensic genetics, genetic polymorphism, Hainan Han, STR

## Abstract

The forensic characteristics and genetic relationships of Hainan Han population are still not fully understood. The aim of this study was to investigate the forensic features and genetic variations of 23 short tandem repeat (STR) included in the Huaxia^TM^ Platinum system in Hainan Han and analyze the population genetic relationships between Hainan Han and other adjacent Chinese populations. The genetic polymorphisms of 23 STR loci included in the Huaxia^TM^ Platinum kit were evaluated from 2971 Hainan Han individuals. Comprehensive comparisons were conducted based on genetic distance, phylogenetic tree, multidimensional scaling and principal component analysis (PCA) to explore inter-population genetic relationship. The combined power of discrimination (CPD) and the combined power of exclusion (CPE) of the 23 STR loci was 0.999 999 999 999 999 999 999 999 999 819 and 0.999 999 999 625 408, respectively. The investigated Hainan Han population has high genetic similarity with geographically close Han populations, while great genetic difference with other ethnic minorities, prominently in Yunnan Miao, Xinjiang Uygurs, Xinjiang Kazakh, and Tibetans. Our study found the 23 STR loci were highly polymorphic and suitable for forensic personal identification and paternity testing in Hainan Han population. Genetic similarity widely existed among Han populations from different regions, and significant genetic divergence existed between Han populations and some ethnic minorities. The populations genetic diversity and similarity were closely associated with ethnic origin and geographical distribution.

## 1. Introduction

The Han Chinese is the ethnic group with the largest population in China and throughout the world. The Han population were originated from the ancient Huaxia tribes in northern China and gradually expanded southward in the past 2 millennia. The Han group are divided into northern Han and southern Han by the Yangtze River. Genetic studies based on Y-chromosome, mitochondrial and microsatellites showed that genetic differentiation exists between northern Han and southern Han.^[1–4]^ In the southward migration, Han population intermarried with the native ethnic groups and gradually forming the current distribution pattern of Han subgroups are distributed widely and gathered tightly in small areas of China.

Hainan is a province located in the southernmost part of China, delimited from Guangdong by the Qiongzhou Strait in the north, opposite Guangxi and Vietnam by the Beibu Gulf in the west, opposite Taiwan by the South China Sea in the east, and adjacent to the Philippines, Brunei and Malaysia in the South China Sea in the southeast and south. This province is the largest free trade port, the second largest island and the largest tropical treasure-house of China. Palaeomagnetic studies show that Hainan island was almost connected to north Vietnam and Guangxi during the Mesozoic, at about the location of the Beibu Gulf, and geological evolution of Tonkin-Beibu Gulf caused Hainan island’s southeast moment.^[5]^ Hainan island was originally inhabited by the natives of Li minority. During the Song dynasty, about 100,000 Han population southward to northern Hainan and settled down.^[6]^ Aside from aforementioned movement, Han population from Fujian and Guangdong province continuously migrated southwards to Hainan during the 17th to 19th century.^[6]^ According to the seventh national census in 2021, the total population in Hainan has exceeded 10 million, mainly including Han, Li, Miao, Hui and other ethnic groups. Han ethnicity accounts for 84.3% of the total population in Hainan (www.stats.gov.cn). Thus, it is necessary to investigate the genetic variations and population structure of Hainan Han population.

As the second generation of DNA genetic marker, short tandem repeat (STR) plays a key role in personal identification and parentage testing due to its high polymorphism, and has been the mainstream identification technology in forensic genetic laboratories.^[7,8]^ Several previous studies have focused on investigating the allele distribution and variation of autosomal STRs in Chinese nationalities.^[9–12]^ The Huaxia^TM^ Platinum System including 23 autosomal STR loci as well as Amelogenin and Y-InDel was specially developed for Chinese populations to forensic application. Although some of STR data on Hainan Han population have been published before, no data of the 23 STR included in the Huaxia^TM^ Platinum kit.^[6,13]^ Wang et al evaluated the genetic polymorphisms of 23 autosomal STR loci using the Huaxia^TM^ Platinum System in 193 unrelated healthy Hainan Han individuals and other 2 main ethnic groups of China for the first time.^[9]^ However, the population data of the 23 STR in Hainan Han population is still limited. Furthermore, due to the small size of the samples in the previous study, the forensic characteristics and genetic relationships of Hainan Han ethnicity population are still not fully understudied. In this study, we enrolled substantial sample to investigated the genetic diversity in Hainan Han using the 23 autosomal STR loci included in the Huaxia^TM^ Platinum System.

## 2. Materials and methods

### 2.1. Ethics statement and sample preparation

This study was reviewed and approved by the Ethics Committee of Hainan Medical University, China (Approval Number: HYLL-2022-097), and written informed consents were obtained from all voluntary participants. A total of 2971 blood samples of unrelated healthy individuals (523 females and 2448 males) were collected from different districts of the Hainan Province. All volunteers met the following screening criteria: all donors including their parents and grandparents are Han populations; all participants residing in Hainan and without migration in their family history; there is no intermarriage with other ethnic groups within 3 generations; all individuals shared no biologically close relationships with each other. Genomic DNA was extracted using the Chelex-100 method.^[14]^

### 2.2. DNA amplification and genotyping

Twenty-three targeted STR loci (D3S1358, vWA, D16S539, CSF1PO, TPOX, D8S1179, D21S11, D18S51, Penta E, D2S441, D19S433, TH01, FGA, D22S1045, D5S818, D13S317, D7S820, D6S1043, D10S1248, D1S1656, D12S391, D2S1338, and Penta D) included in the Huaxia^TM^ Platinum PCR Amplification kit (Thermo Fisher Scientific, MA, USA) were multi-amplified in a GeneAmp PCR system 9700 (Applied Biosystems, USA) according to the manufacturer’s instructions. The amplified products were then separated and detected using capillary electrophoresis on an ABI 3500 DNA Genetic Analyzer (Applied Biosystems, USA). Genotyping was performed using the allelic ladders provided with the kit and the GeneMapper ID v.3.2 software (Applied Biosystems, Foster City, CA). DNA typing and assignment of nomenclature followed the International Society for Forensic Genetics recommendation.^[15]^ Control DNA (007) and ddH_2_O were used as the positive and negative controls, respectively.

### 2.3. Statistical analysis

**Figure 1.**
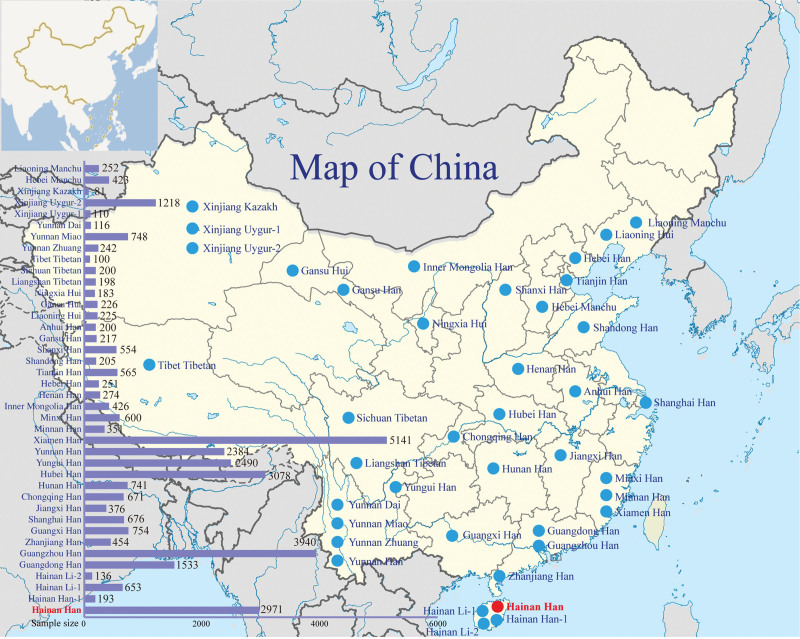
The corresponding geographical location and sample size of the studied Hainan han and other 39 reference populations.

To estimate the forensic efficiency of the 23 STRs in Hainan Han population, genotype data of 2971 individual residents in Hainan were analyzed. The allele frequencies and corresponding forensic parameters including matching probability, power of discrimination, polymorphism information content, probability of exclusion, typical paternity index, expected heterozygosity and observed heterozygosity were evaluated using the Modified-PowerStats v1.2 spread-sheet.^[16]^ Arlequin v3.5.2.2 software was used to estimate the Hardy-Weinberg equilibrium and Linkage disequilibrium (LD).^[17]^ In order to explore the phylogenetic relationships between the studied population and other adjacent groups, the genetic data of 39 Chinese populations (23 Han populations, 3 Hui populations, 3 Tibetan populations, 2 Manchu populations, 2 Li populations, 2 Uygur populations, 1 Kazakh population, 1 Miao population, 1 Zhuang population and 1 Dai population) previously reported were enrolled in our reference data. The geographical location and the detailed information including abbreviation and sample size of these populations are presented in Figure 1 and Table S1, Supplemental Digital Content, http://links.lww.com/MD/M736. Then the standard genetic distances between Hainan Han and other 39 Chinese populations were calculated using Phylip v3.695 and visualized on heatmap.^[18]^ Neighbor-Joining (NJ) phylogenetic tree was constructed based on the genetic distance matrix of the 40 populations using MEGA 7.0 software.^[19]^ Population relationships was further investigated by Multidimensional scaling (MDS), which was conducted using genetic distance and SPSS 21.0 software.^[20]^ Principal component analysis (PCA) was also performed based on allele frequencies using MVSP v3.22.^[21]^

### 2.4. Quality controls

This study was in accordance with the recommendations advocated by the International Society for Forensic Genetics.^[15]^ The experiment was conducted in our forensic genetics laboratory accredited by the China National Accreditation Service for Conformity Assessment. Positive control (007) and negative control (ddH_2_O) were amplification and genotyped along with each batch of samples.

## 3. Results

### 3.1. Genetic diversities and forensic characteristics

All of the 23 autosomal STR loci in this study showed no significant deviations from the Hardy-Weinberg equilibrium (Table S2, Supplemental Digital Content, http://links.lww.com/MD/M737). LD based on loci-by-loci comparisons of 23 autosomal STR loci showed that 13 pairs with a *P* value <.05 (Table S3, Supplemental Digital Content, http://links.lww.com/MD/M738). No association was noted in the analysis of LD after Bonferroni correction. The distribution of allele frequencies and forensic statistical parameters in the Hainan Han population are shown in Table S2, Supplemental Digital Content, http://links.lww.com/MD/M737. A total of 325 alleles and 1464 genotypes for all these loci were found, and 8 (TPOX) to 26 (Penta E and FGA) alleles for each locus were observed. The observed heterozygosity ranged from 0.098 (Penta E) to 0.4217 (TPOX), and the polymorphism information content ranged from 0.5388 (TPOX) to 0.8984 (Penta E). The probability of exclusion ranged from 0.2656 (TPOX) to 0.7995 (Penta E) with a value of 0.999 999 999 625 408 for the combined power of exclusion (CPE). The power of discrimination value ranged from 0.7821 (TPOX) to 0.9836 (Penta E) with a value of 0.999 999 999 999 999 999 999 999 999 819 for the combined power of discrimination (CPD). The typical paternity index and matching probability ranged from 1.1856 (TPOX) to 5.1031 (Penta E), and 0.0164 (Penta E) to 0.2179 (TPOX), respectively.

### 3.2. Comprehensive population comparison

#### 3.2.1. Population standard genetic distances

In order to explore the population genetic diversity, population comparisons were analyzed based on the allele frequencies of 19 shared loci among the 40 populations (geographical location of these populations is presented in Fig. 1). We firstly calculated the Nei’s genetic distance among the 40 populations. As shown in Table S4, Supplemental Digital Content, http://links.lww.com/MD/M739 and Figure S1, Supplemental Digital Content, http://links.lww.com/MD/M749, the Nei’s standard genetic distances ranged from 0.0008 (Yunnan Han and Hubei Han) to 0.1792 (Yunnan Miao and Xinjiang Kazakh) among the 40 populations. Our studied population, Hainan Han had the largest genetic distance with Yunnan Miao (0.0834), followed by Xinjiang Uygur-1 (0.0632) and Xinjiang Kazakh (0.0558), while had the smallest distance with Guangzhou Han (0.001048), followed by Zhanjiang Han (0.0031) and Minxi Han (0.0037).

#### 3.2.2. Phylogenetic relationship analysis

**Figure 2.**
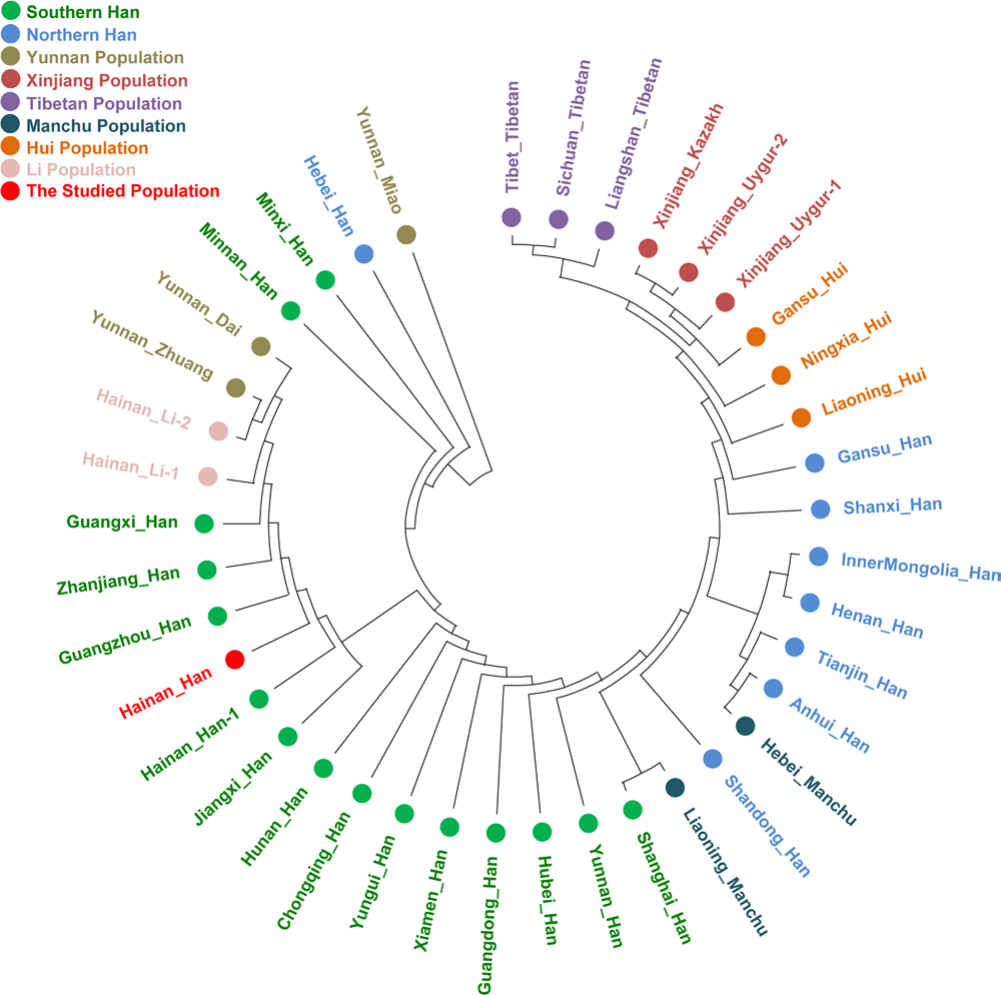
The phylogenetic tree among the 40 populations based on Nei’s genetic distances.

The population relationships among the 40 populations are depicted using a NJ tree based on Nei’s genetic distance matrix. Figure 2 shows the results of NJ phylogenetic tree. As shown in the Figure 2, the Han populations are homogeneously clustered together and substantially different from most of ethnic minorities, especially significant in Yunnan Miao, Xinjiang Kazakh, Xinjiang Uygurs, Tibetan populations, Hui populations, Zhuang and Dai from Yunnan and Lis from Hainan. The NJ tree also shows that populations from the same region (such as Xinjiang Kazakh and 2 Xinjiang Uygurs, Yunnan Zhuang and Yunnan Dai) and the same ethnic group (such as 3 Tibetan populations, 3 Hui populations, and most Han populations) tended to cluster together. Among the Han populations, there are subtle differences between Southern Han and Northern Han and are clustered together respectively. The studied Hainan Han population was observed to cluster closest to the neighboring Han populations, such as Guangzhou Han, Zhanjiang Han, and Guangxi Han, while far away from other ethnic minorities, especially Yunnan Miao, Xinjiang Kazaka, and Uygurs, Tibetans and Huis. Among the 16 ethnic minorities, Hainan Li, Yunnan Zhuang, and Yunnan Dai were tend to clustered close to the studied Hainan Han population.

#### 3.2.3. Multidimensional scaling analysis

**Figure 3.**
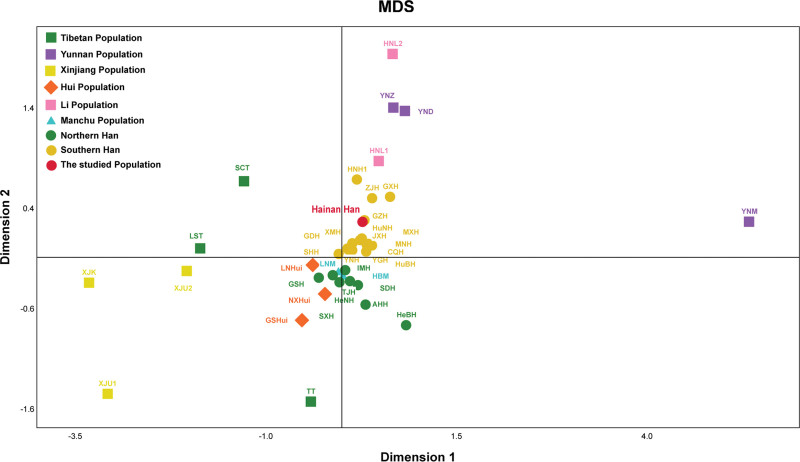
The multidimensional scaling (MDS) plot among the 40 populations based on Nei’s genetic distances. MDS = multidimensional scaling.

Genetic homogeneity and heterogeneity among the 40 populations are further reconstructed using MDS based on the genetic distance matrix. As shown in Figure 3, the similar population which holds a close geographic distance with each other was represented by same label. From the results of MDS, substantial distinction between Han populations and other ethnic minorities, including Uygurs, Kazakh, Tibetans, Miao, Zhuang, Dai, Huis and Lis, were easy observed. In the plots draw, all Han populations are gathered in the center, with other minority groups scattered around the periphery. Slight difference also can be observed between southern Han and northern Han, with the southern Han groups and northern Han groups were clustered together on both sides of the X-axis respectively. The studied Hainan Han population closed to the adjacent Han groups of Guangzhou Han, Zhanjiang Han and Guangxi Han, and great differentiate with other ethnic minorities.

#### 3.2.4. Principal component analysis

**Figure 4.**
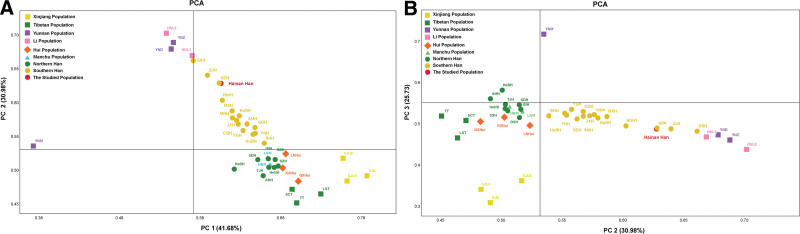
The principal component analysis (PCA) plot based on allele frequencies of 19 shared loci in the 40 Chinese populations. (A) PCA established based on the variance of the first and second components. (B) PCA constructed based on the second and the third components. PCA = principal component analysis.

To further illustrate the genetic relationships between Hainan Han and 39 reference populations. PCA was also estimated based on the normalized allele frequencies to synthetically analyze genetic distance and relatedness between the populations, and PC1, PC2 and PC3 were used to plot a scatter diagram (Fig. 4). The results showed that the first 3 components accounted for 98.39% of the total variance. And the first, second and third principal components interpreted 41.68%, 30.98%, and 25.75%, respectively (Fig. 4). According to the plot of PC12 (Fig. 4A) and PC23 (Fig. 4B), we all observed a clear separation between ethnic minorities and Han populations, as well as smaller diversity between southern Han and northern Han. In the Figure 4A, all southern Han populations were gathered above the X-axis, and all northern Han populations below the X-axis. In the Figure 4B, all southern Han populations were clustered on the right of the Y-axis, and northern Han populations on the left. The investigated Hainan Han population keeps the minimum difference with Guangzhou Han, Zhanjiang Han, and Guangxi Han, significant difference with other ethnic minorities. Among the 16 ethnic minorities, Hainan Han population were tend to close with the geographically adjacent ethnic minority, such as Lis from Hainan, Dai, and Zhuang from Yunnan.

The aforementioned results including the Nei’s genetic distances, NJ tree, MDS and PCA were all demonstrated that Hainan Han have genetically closer relationships with the geographically close Han populations of Guangzhou Han, Zhanjiang Han, and Guangxi Han, while differentiate significantly with other ethnic minority populations, especially with Miao, Uygurs, Kazakh, Tibetans, and Huis. Compared with other ethnic minorities, Hainan Lis, Yunnan Dai and Yunnan Zhuang were close to Hainan Han population.

## 4. Discussion

### 4.1. Genetic polymorphisms and forensic features

China has a total of 56 different ethnic groups with a population of 1.4 billion people. The Han group has the greatest population in China. Owning to the complex demographics and geographical characteristics, genetics research of Chinese populations from different ethnic and/or regional population are of great interest. Although some previous researches about Hainan Han population were reported, the genetic variation and background in Hainan Han population is not fully understood.^[6,9,13]^ The classical genetic marker of STR is widely used in the forensic practice. In this study, a total of 2971 Hainan Han individual are genotyped using 23 STR loci included in the Huaxia^TM^ Platinum kit. All the 23 STR loci were found to be highly genetically polymorphic in the present study. The CPE and CPD are the 2 important indexes to evaluate the forensic efficiency in paternity testing and forensic individual identification. The high value of CPE (0.999 999 999 625 408) and CPD (0.999 999 999 999 999 999 999 999 999 819) indicated that the 23 STR have high forensic efficiency in forensic personal identification and paternity testing in Hainan Han population and can be used as a powerful tool for forensic practice. In addition, this study provides the batch of genetic diversity data of the 23 STR in Hainan Han and can also enriches the Chinese STR reference databases and be useful for other population genetics and diversity studies.

### 4.2. Genetic similarities and differences

To further explore the Chinese population affiliations, the phylogenetic relationship between the studied population and other adjacent Han populations or minority ethnic groups were evaluated. The standard genetic distance indicated that significant genetic differences existed between Han populations and other ethnic minorities, and genetic similarity widely existed among Han populations from different regions. The studied Hainan Han population have more close genetic distance with the adjacent Han populations, such as Guangzhou Han, Zhanjiang Han, and Guangxi Han, and keep a far distance with other ethnic minorities, such as Yunnan Miao, Xinjiang Uygurs, Xinjiang Kazakh, and Tibetans.

The phylogenetic tree revealed that all Han, Hui, Tibetan, Li, and Uygur populations clustered together, respectively. Which indicated the same ethnic populations maybe share more genetic background. We also observed populations from the neighbor regions were tend to gathered close, such as southern Hans, northern Hans, Xinjiang populations and populations from Hainan were gathered respectively. This phenomenon could be explained by that populations living in the same or close geographical position may have high level of gene flow owning to intermarriage. The NJ tree demonstrated that populations from the same ethnic and/or the same geographic area may be more homogeneity. These genetic features are accordance with previous studies using different genetic markers, such as single nucleotide polymorphism, InDel and Microhaplotype.^[22–25]^ According to the MDS, 10 minorities (Yunnan Miao, Xinjiang Kazakh, 2 Xinjiang Uygurs, Tibet Tibetan, Liangshan Tibetan, Sichuang Tibetan, Hainan Li, Yunnan Dai, Yunnan Zhuang) were isolated on the periphery of the MDS scatter diagrams, and Hui, Manchu and Han populations were gathered in the center. Hui and Manchu populations tend to partially overlap with Han populations, which implying the possible genetic admixture between Han and Hui and Manchu populations.

The PCA plot based on allele frequencies were similar with the MDS results based on genetic distance matrix. Substantial genetic distinction exist between Han populations and most ethnic minorities. Aforementioned results reveal that Han populations distributed in different geographic positions have a strong genetic similarity, which indicated that Han populations shares the common ancestry. However, subtle genetic diversity between northern Han and southern Han also observed in the plot of PCA and MDS, which also in accordant with the North-South genetic distinction results of previous researches based on Y chromosomal and mitochondrial DNA.^[2,26,27]^ The results of this comprehensive population comparison revealed that significant genetic differences are identified between Hainan Han population and some minority ethnic groups, especially in Yunnan Miao, Xinjiang Uygurs, Xinjiang Kazakh, and Tibetans, which is similar with previous research.^[9]^ These genetic differences may be related with that Qiongzhou Strait separates Hainan island from the continent, acts as a significant barrier to gene flow. Besides, ethnic origin, geographical distribution, social and culture background also play roles in genetic distinction. Compared to other minorities, Hainan Li, Yunnan Dai and Yunnan Zhuang keep a closer genetic relationship with our investigated population. Which may be relate with that Hainan island had drifted southward from a more northwestern position. Among the Han populations, the studied Hainan Han were a strong genetic affinity with the adjacent Han populations, especially with Guangzhou Han, Zhanjiang Han, Guangxi Han, which may be relate with large scale southward migration of Han population in historic period. In the southward migration, Han population intermarried with the native Li population, which could be explained why Hainan Han have closer relationship with Hainan Li than other ethnic minorities.

## 5. Conclusion

In summary, we obtained the genotype data of the 23 STR included in the Huaxia^TM^ Platinum kit in Hainan Han population, which can enrich the database of the Hainan Han population in China. The allele frequencies and forensic parameters suggested that these 23 STR are highly informative in Hainan Han and can be used in forensic application such as paternity testing, individual identification, and genetic population studies. The population comprehensive comparison based on the Nei’s genetic distances, NJ tree, MDS and PCA illustrated that significant genetic differences existed between Han populations and some ethnic minorities, and genetic similarity widely existed among Han populations from different regions. Besides, the subtle North-South genetic differentiation was also observed. The inter-population comparison consistently indicated that the investigated Hainan Han have high genetic affinity with the geographically close Han populations, and keep distinct relationship with other ethnic minorities, prominently in Yunnan Miao, Xinjiang Uygurs, Xinjiang Kazakh, and Tibetans. Which also implied that populations genetic diversity and affinity are closely associated with ethnic origin, population migration and geographical distribution.

## Acknowledgments

The authors are very grateful to all sample donors for their contributions to this work and all those who helped with sample collection.

## Author contributions

**Data curation:** Qianyun Nie.

**Formal analysis:** Wenhui Li, Tao Song.

**Software:** Yinyu Chen.

**Writing – original draft:** Xing Zou.

**Writing – review & editing:** Peng Zhang.

## Supplementary Material









**Figure:**