# Supplementary material for: Genetic variation and phylogenetic analysis of 23 STR in Chinese Han population from Hainan, Southern China
Source: Medicine (Baltimore). 2024 May 31;103(22):e38428. doi: 10.1097/MD.0000000000038428 (PMC11142786; doi:10.1097/MD.0000000000038428)
Supplement: Supplementary file 1 [file medi-103-e38428-s001.pdf]

Table S1. The details of relevant populations.

Population name	Abbreviation	Sample size
Hainan Han	HNH	2971
Hainan Han-1	HNH1	193
Hainan Li-1	HNL1	653
Hainan Li-2	HNL2	136
Guangdong Han	GDH	1533
Guangzhou Han	GZH	3940
Zhanjiang Han	ZJH	454
Guangxi Han	GXH	754
Shanghai Han	SHH	676
Jiangxi Han	JXH	376
Chongqing Han	CQH	671
Hunan Han	HuNH	741
Hubei Han	HuBH	3078
Yungui Han	YGH	2490
Yunnan Han	YNH	2384
Xiamen Han	XMH	5141
Minnan Han	MNH	351
Minxi Han	MXH	600
Inner Mongolia Han	IMH	426
Henan Han	HeNH	274
Hebei Han	HeBH	251
Tianjin Han	TJH	565
Shandong Han	SDH	205
Shanxi Han	SXH	554
Gansu Han	GSH	217
Anhui Han	AHH	200
Liaoning Hui	LNHui	225
Gansu Hui	GSHui	226
Ningxia Hui	NXHui	183
Liangshan Tibetan	LST	198
Sichuan Tibetan	SCT	200
Tibet Tibetan	TT	100
Yunnan Zhuang	YNZ	242
Yunnan Miao	YNM	748
Yunnan Dai	YND	116
Xinjiang Uygur-1	XJU1	110
Xinjiang Uygur-2	XJU2	1218
Xinjiang Kazakh	XJK	81
Hebei Manchu	HBM	423
Liaoning Manchu	LNM	252
