# Supplementary material for: Genetic variation and phylogenetic analysis of 23 STR in Chinese Han population from Hainan, Southern China
Source: Medicine (Baltimore). 2024 May 31;103(22):e38428. doi: 10.1097/MD.0000000000038428 (PMC11142786; doi:10.1097/MD.0000000000038428)
Supplement: Supplementary file 2 [file medi-103-e38428-s002.pdf]

Table S2. Allele frequencies and statistical parameters of the 23 STR loci in Chinese Han population from Hainan (n=2971).

Allele	D3S1358	vWA	D16S539	CSF1PO	TPOX	D8S1179	D21S11	D18S51	Penta E	D2S441	D19S433	TH01
5	-	-	-	-	0.0002	-	-	-	0.0483	-	-	0.0007
6	-	-	-	-	-	-	-	-	0.0002	-	-	0.1010
7	-	-	-	0.0076	0.0008	-	-	0.0002	0.0007	-	-	0.3017
8	-	-	0.0042	0.0008	0.5574	0.0007	-	-	0.0027	0.0003	-	0.0554
9	-	-	0.2550	0.0330	0.1133	0.0008	-	0.0007	0.0141	0.0027	0.0010	0.4589
9.1	-	-	-	-	-	-	-	-	0.0002	0.0246	-	0.0002
9.3	-	-	-	-	-	-	-	-	-	-	-	0.0296
10	-	-	0.1294	0.2279	0.0278	0.1367	-	0.0002	0.0439	0.2178	-	0.0513
10.1	-	-	-	-	-	-	-	-	-	0.0024	-	-
10.3	-	-	-	-	-		-	-	-	0.0002	-	-
11	-	0.0001	0.2816	0.2543	0.2782	0.1162	-	0.0051	0.1936	0.3144	0.0022	0.0012
11.1	-	-	-	-	-	-	-	-	-	0.0002	-	-
11.2	-	-	-	-	-	-	-		-	0.0003	-	-
11.3	-	-	-	-	-	-	-	-	0.0005	0.0789	-	-
12	0.0007	-	0.2201	0.3863	0.0215	0.1312	-	0.0455	0.1340	0.1779	0.0411	-
12.1	-	-	-	-	-	-	-	-	-	-	-	-
12.2	-	-	-	-	-	-	-	-	-	-	0.0040	-
12.3	-	-	-	-	-	-	-	-	-	0.0008	-	-
13	0.0040	0.0003	0.0929	0.0781	0.0008	0.1911	-	0.1577	0.0571	0.0210	0.2874	-
13.2	-	-	-	-	-	-	-	-	-	-	0.0293	-
14	0.0389	0.2910	0.0162	0.0101	-	0.1639	-	0.1983	0.0793	0.1486	0.2458	-
14.2	-	-	-	-	-	-	-	-	-	0.0005	0.1010	-
15	0.3235	0.0195	0.0003	0.0017	-	0.1701	-	0.1891	0.0791	0.0084	0.0721	-
15.2	-	-	-	-	-	-	-	-	0.0002	-	0.1600	-
15.3	-	-	-	-	-	-	-	-	-	-	-	-
16	0.3133	0.1547	0.0003	0.0002	-	0.0691	-	0.1448	0.0663	0.0010	0.0182	-
16.2	-	-	-	-	-	-	-	-	-	-	0.0330	-
16.3	-	-	-	-	-		-	-	-	-	-	-
17	0.2511	0.2335	-	-	-	0.0167	-	0.0800	0.0751	-	0.0013	-
17.2	-	-	-	-	-	-	-	-	-	-	0.0029	-
17.3	-	0.0002	-	-	-	-	-	-	-	-	-	-
18	0.0606	0.1988	-	-	-	0.0035	-	0.0586	0.0662	-	-	-
18.2	-	-	-	-	-	-	-	-	-	-	0.0007	-
18.3	-	-	-	-	-	-	-	-	-	-	-	-
18.4	-	-	-	-	-	-	-	-	0.0010	-	-	-
19	0.0076	0.0848	-	-	-	-	-	0.0426	0.0455	-	-	-
19.3	-	-	-	-	-	-	-	-	-	-	-	-
20	0.0003	0.0151	-	-	-	-	-	0.0251	0.0397	-	-	-
20.2	-	-	-	-	-	-	-	-	-	-	-	-
20.3	-	-	-	-	-	-	-	-	-	-	-	-
21	-	0.0020	-	-	-	-	-	0.0204	0.0212	-	-	-
21.2	-	-	-	-	-	-	-	-	-	-	-	-
21.3	-	-	-	-	-	-	-	-	-	-	-	-
22	-	-	-	-	-	-	-	0.0192	0.0128	-	-	-
22.2	-	-	-	-	-	-	-	-	-	-	-	-
23	-	-	-	-	-	-	-	0.0071	0.0093	-	-	-
23.1	-	-	-	-	-	-	-	-	-	-	-	-
23.2	-	-	-	-	-	-	-	-	-	-	-	-
24	-	-	-	-	-	-	-	0.0037	0.0059	-	-	-
24.2	-	-	-	-	-	-	-	-	-	-	-	-
25	-	-	-	-	-	-	-	0.0015	0.0019	-	-	-
25.2	-	-	-	-	-	-	-	-	-	-	-	-
26	-	-	-	-	-	-	0.0005	0.0002	0.0012		-	-
26.2	-	-	-	-	-	-	-	-	-	-	-	-
27	-	-	-	-	-	-	0.0022	-	-	-	-	-
27.2	-	-	-	-	-	-	-	-	-	-	-	-
28	-	-	-	-	-	-	0.0574	-	-	-	-	-
28.1	-	-	-	-	-	-	0.0002	-	-	-	-	-
28.2	-	-	-	-	-	-	0.0015	-	-	-	-	-
29	-	-	-	-	-	-	0.2616	-	-	-	-	-
29.2	-	-	-	-	-	-	0.0010	-	-	-	-	-
30	-	-	-	-	-	-	0.2530	-	-	-	-	-
30.2	-	-	-	-	-	-	0.0080	-	-	-	-	-
30.3	-	-	-	-	-	-	0.0024	-	-	-	-	-
31	-	-	-	-	-	-	0.0810	-	-	-	-	-
31.1	-	-	-	-	-	-	0.0002	-	-	-	-	-
31.2	-	-	-	-	-	-	0.0828	-	-	-	-	-
32	-	-	-	-	-	-	0.0340	-	-	-	-	-
32.2	-	-	-	-	-	-	0.1542	-	-	-	-	-
33	-	-	-	-	-	-	0.0040	-	-	-	-	-
33.2	-	-	-	-	-	-	0.0508	-	-	-	-	-
34	-	-	-	-	-	-	0.0008	-	-	-	-	-
34.1	-	-	-	-	-	-	0.0002	-	-	-	-	-
34.2	-	-	-	-	-	-	0.0039	-	-	-	-	-
35.2	-	-	-	-	-	-	0.0003	-	-	-	-	-
MP	0.1223	0.0751	0.0813	0.1187	0.2179	0.0398	0.0531	0.0344	0.0164	0.0714	0.0586	0.1479
PD	0.8777	0.9249	0.9187	0.8813	0.7821	0.9602	0.9468	0.9656	0.9836	0.9287	0.9414	0.8521
PIC	0.6799	0.7577	0.7471	0.6813	0.5388	0.8355	0.8015	0.8491	0.8984	0.7632	0.7878	0.6330
PE	0.4757	0.5436	0.5430	0.4658	0.2656	0.6955	0.6313	0.7503	0.7995	0.5622	0.5810	0.3781
TPI	1.8499	2.1686	2.1655	1.8094	1.1856	3.3401	2.7348	4.0909	5.1031	2.2714	2.3836	1.4985
HO	0.2703	0.2306	0.2309	0.2763	0.4217	0.1497	0.1828	0.1222	0.0980	0.2201	0.2098	0.3337
HE	0.7297	0.7694	0.7691	0.7237	0.5783	0.8503	0.8172	0.8778	0.9020	0.7799	0.7902	0.6663
X2	0.0079	7.3928	2.7801	0.1548	4.8000	0.2259	0.7956	4.8762	0.4376	3.0182	9.2798	3.2356
p	0.9292	0.0065	0.0954	0.6940	0.0285	0.6345	0.3724	0.0272	0.5083	0.0823	0.0023	0.0721

Allele	FGA	D22S1045	D5S818	D13S317	D7S820	D6S1043	D10S1248	D1S1656	D12S391	D2S1338	Penta D
5	-	-	-	0.0005	-	-	-	-	-	-	-
6	-	-	-	-	0.0003	-	-	-	-	-	0.0010
7	-	-	0.0416	0.0020	0.0064	-	-	-	-	-	0.0138
8	-	-	0.0034	0.3156	0.1562	-	0.0015	-	-	-	0.0665
9	-	-	0.0717	0.1405	0.0668	0.0017	-	0.0003	-	-	0.3712
9.1	-	-	-	-	0.0030	-	-	-	-	-	-
9.3	-	-	-	-	0.0003	-	-	-	-	-	-
10	-	0.0003	0.2070	0.1560	0.1606	0.0306	0.0007	0.0007	-	-	0.1375
10.1	-	-	-	-	0.0007	-	-	-	-	-	-
10.3	-	-	-	-	-	-	-	-	-	-	-
11	-	0.1723	0.2867	0.2274	0.3533	0.1247	0.0027	0.0800	-	-	0.1192
11.1	-	-	-	-	0.0007	-	-	-	-	-	-
11.2	-	-	-	-	-	-	-	-	-	-	-
11.3	-	-	-	-	-	-	-	-	-	-	-
12	-	0.0027	0.2311	0.1222	0.2140	0.1321	0.0677	0.0411	-	-	0.1373
12.1	-	0.0002	-	-	-	-	-	-	-	-	-
12.2	-	-	-	-	-	-	-	-	-	-	-
12.3	-	-	-	-	-	0.0010	-	-	-	-	-
13	0.0037	0.0045	0.1476	0.0301	0.0337	0.1211	0.3418	0.0994	-	-	0.0946
13.2	-	-	-	-	-	-	-	-	-	-	-
14	0.0003	0.0402	0.0094	0.0057	0.0037	0.1321	0.2291	0.0948	-	0.0002	0.0446
14.2	-	-	-	-	-	-	-	0.0002	-	-	-
15	0.0003	0.3185	0.0015	-	0.0003	0.0188	0.2263	0.2868	0.0128	-	0.0126
15.2	-	-	-	-	-	-	-	-	-	-	-
15.3	-	-	-	-	-	-	-	0.0008	-	-	-
16	0.0019	0.2257	-	-	-	0.0050	0.1044	0.1927	0.0035	0.0125	0.0015
16.2	-	-	-	-	-	-	-	-	-	-	-
16.3	-	-	-	-	-	-	-	0.0051	-	-	-
17	0.0010	0.2073	-	-	-	0.0353	0.0226	0.0751	0.0752	0.0793	0.0002
17.2	-	-	-	-	-	-	-	0.0002		-	-
17.3	-	-	-	-	-	0.0008	-	0.0777	0.0003	-	-
18	0.0256	0.0256	-	-	-	0.1750	0.0032	0.0142	0.1988	0.0922	-
18.2	-	-	-	-	-	0.0003	-	-	-	-	-
18.3	-	-	-	-	-	-	-	0.0270	-	-	-
18.4	-		-	-	-	-	-	-	-	-	-
19	0.0732	0.0025	-	-	-	0.1463	-	0.0017	0.1934	0.2024	-
19.3	-	-	-	-	-	0.0002	-	0.0022	0.0005		-
20	0.0446	0.0002	-	-	-	0.0645	-	-	0.2001	0.1186	-
20.2	0.0002	-	-	-	-	-	-	-	-	-	-
20.3	-	-	-	-	-	0.0005	-	-	-	-	-
21	0.1262	-	-	-	-	0.0079	-	-	0.1274	0.0335	-
21.2	0.0034	-	-	-	-	-	-	-	-	-	-
21.3	-	-	-	-	-	0.0003	-	-	-	-	-
22	0.1809	-	-	-	-	0.0013	-	-	0.0947	0.0456	-
22.2	0.0061	-	-	-	-	-	-	-	-	-	-
23	0.1998	-	-	-	-	0.0005	-	-	0.0525	0.1733	-
23.1	0.0002	-	-	-	-	-	-	-	-	-	-
23.2	0.0077	-	-	-	-	-	-	-	-	-	-
24	0.1609	-	-	-	-	-	-	-	0.0264	0.1590	-
24.2	0.0082	-	-	-	-	-	-	-	-	-	-
25	0.0941	-	-	-	-	-	-	-	0.0109	0.0730	-
25.2	0.0057	-	-	-	-	-	-	-	-	-	-
26	0.0407	-	-	-	-	-	-	-	0.0032	0.0099	-
26.2	0.0029	-	-	-	-	-	-	-	-	-	-
27	0.0096	-	-	-	-	-	-	-	0.0003	0.0003	-
27.2	0.0003	-	-	-	-	-	-	-	-	-	-
28	0.0022	-	-	-	-	-	-	-	-	0.0002	-
28.1	-	-	-	-	-	-	-	-	-	-	-
28.2	-	-	-	-	-	-	-	-	-	-	-
29	0.0003	-	-	-	-	-	-	-	-	-	-
29.2	-	-	-	-	-	-	-	-	-	-	-
30	-	-	-	-	-	-	-	-	-	-	-
30.2	-	-	-	-	-	-	-	-	-	-	-
30.3	-	-	-	-	-	-	-	-	-	-	-
31	-	-	-	-	-	-	-	-	-	-	-
31.1	-	-	-	-	-	-	-	-	-	-	-
31.2	-	-	-	-	-	-	-	-	-	-	-
32	-	-	-	-	-	-	-	-	-	-	-
32.2	-	-	-	-	-	-	-	-	-	-	-
33	-	-	-	-	-	-	-	-	-	-	-
33.2	-	-	-	-	-	-	-	-	-	-	-
34	-	-	-	-	-	-	-	-	-	-	-
34.1	-	-	-	-	-	-	-	-	-	-	-
34.2	-	-	-	-	-	-	-	-	-	-	-
35.2	-	-	-	-	-	-	-	-	-	-	-
MP	0.0318	0.0890	0.0736	0.0755	0.0836	0.0286	0.0929	0.0424	0.0414	0.0323	0.0642
PD	0.9682	0.9110	0.9264	0.9245	0.9164	0.9714	0.9071	0.9576	0.9586	0.9677	0.9358
PIC	0.8526	0.7366	0.7618	0.7580	0.7413	0.8633	0.7266	0.8240	0.8305	0.8517	0.7723
PE	0.7240	0.5574	0.5787	0.5701	0.5676	0.7463	0.5399	0.6824	0.6973	0.7066	0.5677
TPI	3.6953	2.2440	2.3692	2.3175	2.3023	4.0257	2.1491	3.1983	3.3609	3.4708	2.3031
HO	0.1353	0.2228	0.2110	0.2158	0.2172	0.1242	0.2327	0.1563	0.1488	0.1441	0.2171
HE	0.8647	0.7772	0.7890	0.7842	0.7828	0.8758	0.7673	0.8437	0.8512	0.8559	0.7829
X2	0.1198	0.3219	0.2808	0.3877	1.4046	0.0072	0.2397	0.1588	0.1542	2.7137	2.5067
p	0.7293	0.5705	0.5962	0.5337	0.2360	0.9326	0.6245	0.6902	0.6945	0.0995	0.1134

STR: short tandem repeat;

MP: matching probability;

PD: power of discrimination;

PIC: polymorphism information content;

PE: probability of exclusion;

TPI: typical paternity index;

HO: observed heterozygosity;

HE: expected heterozygosity;

P: probability values of exact tests for Hardy-Weinberg equilibrium.
