# Supplementary material for: Genetic variation and phylogenetic analysis of 23 STR in Chinese Han population from Hainan, Southern China
Source: Medicine (Baltimore). 2024 May 31;103(22):e38428. doi: 10.1097/MD.0000000000038428 (PMC11142786; doi:10.1097/MD.0000000000038428)
Supplement: Supplementary file 3 [file medi-103-e38428-s003.pdf]

Table S3. The p-values of LD between all 23 STR loci from Hainan Han population.

Loci	D3S1358	vWA	D16S539	CSF1PO	TPOX	D8S1179	D21S11	D18S51	Penta E	D2S441	D19S433	TH01	FGA	D22S1045	D5S818	D13S317	D7S820	D6S1043	D10S1248	D1S1656	D12S391	D2S1338
vWA	0.8387																					
D16S539	0.2199	0.0645																				
CSF1PO	0.3998	0.8123	0.6432																			
TPOX	0.9462	**0.0137**	0.8348	0.9717																		
D8S1179	0.1916	0.8592	0.8983	0.5503	0.4164																	
D21S11	0.8553	0.4321	0.3597	0.0772	0.6618	0.7038																
D18S51	0.3021	0.3099	0.1212	0.1505	0.7840	0.1251	0.7947															
Penta E	0.0772	0.5992	0.6110	0.1545	0.7028	0.6364	0.2590	0.2346														
D2S441	0.9238	0.4018	0.3773	0.1222	0.4849	0.3451	0.0841	0.2043	0.1789													
D19S433	0.2219	0.6530	0.7762	0.5269	0.1789	0.4868	0.3529	0.3734	0.1212	0.9550												
TH01	0.1740	0.4585	0.2923	0.4966	0.1975	**0.0127**	0.1730	0.7752	0.2209	0.3861	**0.0244**											
FGA	0.6530	0.3519	0.8983	0.5396	0.8876	0.6285	0.6716	0.2082	0.8632	0.8544	0.1173	**0.0391**										
D22S1045	0.4497	0.5112	0.5728	0.7810	0.3412	0.2835	0.5112	0.9023	0.3343	0.6667	0.3148	0.6422	0.6090									
D5S818	0.0684	0.7654	**0.0196**	0.3236	0.1310	0.4467	0.3148	0.5973	0.2209	0.1975	0.8436	0.6080	0.6735	0.2190								
D13S317	0.7097	0.4741	0.3617	0.5719	0.7439	0.9306	0.8622	0.1808	0.6569	0.1095	0.1261	0.2708	0.6452	0.0655	0.9629							
D7S820	0.5631	0.6628	0.5865	0.6755	0.6716	0.6373	0.9286	0.5249	0.4282	0.2718	0.8465	0.5670	0.0694	0.5249	0.2151	0.8915						
D6S1043	0.4780	0.3001	0.0802	0.2571	0.8260	0.3138	0.9140	**0.0323**	0.9189	0.4585	0.1222	0.6227	0.4663	0.0508	0.8377	0.9492	0.1975					
D10S1248	0.2434	0.9844	0.9511	0.8710	0.4741	0.6804	0.1427	0.1329	0.6637	0.4633	0.2053	0.1486	0.3656	0.1349	0.9345	0.2972	0.6393	0.9531				
D1S1656	0.6090	0.6735	0.5914	0.3187	0.6892	0.5650	0.6100	0.7654	0.6931	0.4819	0.3979	0.9922	0.3998	**0.0186**	0.2942	0.8964	0.4106	0.8622	0.3109			
D12S391	0.2395	**0.0049**	0.8416	0.8837	0.6911	0.6921	0.9648	0.4966	0.5836	0.8397	0.3402	0.3402	0.6256	0.9257	0.2072	0.4546	**0.0078**	**0.0362**	0.8436	0.6481		
D2S1338	0.7449	0.8954	0.1906	0.1134	0.7067	0.3754	0.2229	0.3881	0.1574	0.0528	0.3470	0.7038	0.8241	0.4565	0.4438	0.9775	0.2053	0.2551	0.1310	0.6325	0.4487	
Penta D	0.2297	0.8974	**0.0391**	0.6227	0.4643	0.2131	0.1525	0.7390	**0.0469**	0.3343	0.5709	0.0635	0.1906	0.5503	0.7957	0.0743	0.8074	0.1808	0.8100	0.0841	0.8759	**0.0205**

Statistically significant *p* values ($p < 0.05$) are indicated in bold.
