# Supplementary material for: Genetic variation and phylogenetic analysis of 23 STR in Chinese Han population from Hainan, Southern China
Source: Medicine (Baltimore). 2024 May 31;103(22):e38428. doi: 10.1097/MD.0000000000038428 (PMC11142786; doi:10.1097/MD.0000000000038428)
Supplement: Supplementary file 4 [file medi-103-e38428-s004.pdf]

Table S4. The Nei's genetic distance between the Hainan Han population and 39 reference populations.

Population	Hainan Han	Hainan Han -1	Hainan Li -1	Hainan Li -2	Guangdong Ha	Guangzhou Ha	Zhanjiang Har	Guangxi Han	Shanghai Har	Jiangxi Han	Chongqing Ha	Hunan Han	Hubei Han	Yungui Han	Yunnan Han	Xiamen Han	Minnan Han	Minxi Han	ner Mongolia I	Henan Han	Hebei Han	Tianjin Han	Shandong Har	Shanxi Han	Gansu Han	Anhui Han	Liaoning Hui	Gansu Hui	Ningxia Hui	iangshanTibet	Sichuan Tibeta	Tibet Tibetan	'unnan Zhuan	Yunnan Miao	Yunnan Dai	(injiang Uygur	(injiang-Uygur	(injiang Kazak	Hebei Manchu	iaoning Manchu
Hainan Han																																								
Hainan Han -1[1]	0.0105																																							
Hainan Li -1[2]	0.0057	0.0154																																						
Hainan Li -2[3]	0.0177	0.0352	0.0161																																					
Guangdong Han[4]	0.0044	0.0128	0.0133	0.0298																																				
Guangzhou Han[5]	0.0010	0.0107	0.0057	0.0192	0.0043																																			
Zhanjiang Han[5]	0.0031	0.0121	0.0059	0.0215	0.0085	0.0030																																		
Guangxi Han[5]	0.0039	0.0149	0.0056	0.0178	0.0092	0.0033	0.0048																																	
Shanghai Han[6]	0.0076	0.0161	0.0196	0.0362	0.0032	0.0076	0.0116	0.0154																																
Jiangxi Han[5]	0.0075	0.0147	0.0157	0.0322	0.0068	0.0067	0.0098	0.0132	0.0073																															
Chongqing Han[7]	0.0052	0.0135	0.0133	0.0300	0.0029	0.0047	0.0081	0.0096	0.0041	0.0061																														
Hunan Han[5]	0.0045	0.0157	0.0126	0.0258	0.0031	0.0044	0.0083	0.0089	0.0051	0.0073	0.0037																													
Hubei Han[8]	0.0055	0.0129	0.0152	0.0328	0.0019	0.0052	0.0088	0.0117	0.0022	0.0052	0.0030	0.0029																												
Yungui Han[9]	0.0051	0.0134	0.0139	0.0297	0.0022	0.0049	0.0083	0.0103	0.0034	0.0055	0.0023	0.0032	0.0019																											
Yunnan Han[10]	0.0050	0.0122	0.0149	0.0316	0.0015	0.0048	0.0082	0.0110	0.0019	0.0054	0.0028	0.0029	0.0008	0.0015																										
Xiamen Han[11]	0.0037	0.0111	0.0128	0.0297	0.0019	0.0035	0.0066	0.0095	0.0027	0.0047	0.0025	0.0031	0.0010	0.0020	0.0011																									
Minnan Han[12]	0.0069	0.0159	0.0148	0.0309	0.0063	0.0070	0.0092	0.0119	0.0072	0.0080	0.0065	0.0069	0.0060	0.0052	0.0058	0.0051																								
Minxi Han[13]	0.0037	0.0119	0.0110	0.0258	0.0031	0.0032	0.0070	0.0070	0.0056	0.0065	0.0040	0.0033	0.0032	0.0035	0.0032	0.0024	0.0063																							
Inner Mongolia Han[14]	0.0110	0.0173	0.0214	0.0406	0.0043	0.0113	0.0146	0.0177	0.0050	0.0099	0.0059	0.0073	0.0040	0.0045	0.0039	0.0047	0.0087	0.0072																						
Henan Han[15]	0.0136	0.0217	0.0253	0.0443	0.0055	0.0135	0.0172	0.0209	0.0053	0.0121	0.0079	0.0089	0.0051	0.0059	0.0050	0.0063	0.0105	0.0095	0.0043																					
Hebei Han[16]	0.0221	0.0290	0.0331	0.0553	0.0156	0.0225	0.0260	0.0292	0.0160	0.0197	0.0176	0.0176	0.0148	0.0136	0.0147	0.0154	0.0194	0.0163	0.0155	0.0159																				
Tianjin Han[17]	0.0120	0.0180	0.0253	0.0453	0.0052	0.0124	0.0160	0.0211	0.0044	0.0091	0.0057	0.0074	0.0035	0.0042	0.0036	0.0043	0.0102	0.0083	0.0043	0.0051	0.0148																			
Shandong Han[18]	0.0136	0.0189	0.0242	0.0433	0.0082	0.0136	0.0169	0.0219	0.0081	0.0117	0.0091	0.0110	0.0072	0.0074	0.0065	0.0072	0.0122	0.0105	0.0080	0.0085	0.0170	0.0077																		
Shanxi Han[19]	0.0116	0.0168	0.0228	0.0440	0.0050	0.0120	0.0145	0.0205	0.0047	0.0094	0.0061	0.0082	0.0046	0.0047	0.0038	0.0051	0.0098	0.0090	0.0043	0.0043	0.0152	0.0036	0.0071																	
Gansu Han[20]	0.0155	0.0246	0.0270	0.0452	0.0089	0.0163	0.0191	0.0231	0.0096	0.0161	0.0093	0.0099	0.0088	0.0093	0.0077	0.0099	0.0143	0.0126	0.0096	0.0106	0.0236	0.0087	0.0138	0.0082																
Anhui Han[21]	0.0177	0.0238	0.0288	0.0487	0.0088	0.0173	0.0227	0.0251	0.0090	0.0150	0.0103	0.0113	0.0073	0.0087	0.0074	0.0091	0.0156	0.0118	0.0074	0.0081	0.0195	0.0068	0.0115	0.0076	0.0134															
Liaoning Hui[22]	0.0119	0.0178	0.0237	0.0402	0.0059	0.0123	0.0175	0.0208	0.0071	0.0099	0.0088	0.0094	0.0063	0.0072	0.0062	0.0071	0.0113	0.0095	0.0076	0.0086	0.0197	0.0070	0.0101	0.0068	0.0122	0.0123														
Gansu Hui[23]	0.0217	0.0264	0.0352	0.0558	0.0136	0.0217	0.0266	0.0294	0.0128	0.0188	0.0145	0.0171	0.0134	0.0142	0.0132	0.0145	0.0201	0.0174	0.0135	0.0141	0.0264	0.0122	0.0180	0.0120	0.0160	0.0161	0.0139													
Ningxia Hui[24]	0.0166	0.0218	0.0283	0.0451	0.0108	0.0174	0.0218	0.0256	0.0107	0.0173	0.0124	0.0128	0.0102	0.0104	0.0096	0.0110	0.0163	0.0144	0.0099	0.0117	0.0222	0.0101	0.0126	0.0085	0.0134	0.0117	0.0113	0.0155												
Liangshan Tibetan[1]	0.0335	0.0416	0.0466	0.0613	0.0243	0.0336	0.0385	0.0459	0.0252	0.0317	0.0280	0.0293	0.0252	0.0272	0.0242	0.0261	0.0327	0.0315	0.0255	0.0237	0.0413	0.0253	0.0273	0.0204	0.0272	0.0296	0.0225	0.0264	0.0253											
Sichuan Tibetan[24]	0.0324	0.0392	0.0440	0.0561	0.0230	0.0310	0.0365	0.0415	0.0233	0.0312	0.0250	0.0264	0.0230	0.0231	0.0223	0.0232	0.0324	0.0279	0.0217	0.0221	0.0390	0.0229	0.0259	0.0198	0.0265	0.0258	0.0262	0.0286	0.0275	0.0237										
Tibet Tibetan[25]	0.0349	0.0422	0.0477	0.0587	0.0253	0.0354	0.0410	0.0450	0.0260	0.0317	0.0282	0.0300	0.0270	0.0256	0.0262	0.0277	0.0337	0.0296	0.0254	0.0246	0.0334	0.0256	0.0289	0.0216	0.0294	0.0286	0.0257	0.0254	0.0280	0.0272	0.0209									
Yunnan Zhuang[26]	0.0116	0.0240	0.0136	0.0226	0.0195	0.0110	0.0112	0.0087	0.0259	0.0240	0.0206	0.0190	0.0219	0.0207	0.0212	0.0192	0.0213	0.0175	0.0311	0.0331	0.0391	0.0340	0.0339	0.0328	0.0337	0.0392	0.0323	0.0445	0.0403	0.0529	0.0531	0.0558								
Yunnan Miao[27]	0.0834	0.0940	0.0871	0.0946	0.0842	0.0823	0.0861	0.0775	0.0906	0.0873	0.0792	0.0817	0.0841	0.0760	0.0853	0.0851	0.0795	0.0762	0.0879	0.0919	0.0745	0.0873	0.0891	0.0931	0.1023	0.0842	0.0992	0.1064	0.0965	0.1442	0.1232	0.1103	0.0911							
Yunnan Dai[26]	0.0133	0.0238	0.0174	0.0255	0.0212	0.0123	0.0143	0.0133	0.0241	0.0222	0.0190	0.0187	0.0219	0.0210	0.0216	0.0195	0.0244	0.0178	0.0335	0.0359	0.0399	0.0325	0.0339	0.0322	0.0351	0.0364	0.0328	0.0454	0.0375	0.0619	0.0492	0.0587	0.0167	0.0911						
Xinjiang Uygur-1[24]	0.0632	0.0660	0.0717	0.0944	0.0536	0.0635	0.0685	0.0709	0.0553	0.0639	0.0621	0.0599	0.0567	0.0579	0.0545	0.0579	0.0624	0.0590	0.0572	0.0564	0.0696	0.0591	0.0599	0.0528	0.0552	0.0616	0.0465	0.0553	0.0535	0.0704	0.0727	0.0658	0.0848	0.1664	0.0835					
Xinjiang Uygur-2[28]	0.0367	0.0471	0.0481	0.0616	0.0300	0.0374	0.0439	0.0432	0.0316	0.0388	0.0359	0.0331	0.0323	0.0330	0.0318	0.0334	0.0353	0.0345	0.0319	0.0331	0.0506	0.0345	0.0426	0.0329	0.0350	0.0392	0.0270	0.0283	0.0330	0.0424	0.0467	0.0414	0.0550	0.1420	0.0605	0.0348				
Xinjiang Kazakh[29]	0.0558	0.0691	0.0704	0.0822	0.0483	0.0564	0.0658	0.0653	0.0495	0.0613	0.0570	0.0544	0.0525	0.0551	0.0514	0.0535	0.0604	0.0546	0.0506	0.0530	0.0711	0.0526	0.0611	0.0506	0.0575	0.0596	0.0437	0.0473	0.0478	0.0522	0.0651	0.0602	0.0821	0.1792	0.0858	0.0581	0.0240			
Hebei Manchu[30]	0.0133	0.0191	0.0251	0.0437	0.0060	0.0132	0.0167	0.0214	0.0048	0.0114	0.0070	0.0080	0.0044	0.0050	0.0042	0.0053	0.0100	0.0087	0.0051	0.0054	0.0164	0.0046	0.0078	0.0052	0.0095	0.0062	0.0084	0.0135	0.0099	0.0244	0.0234	0.0259	0.0322	0.0919	0.0326	0.0569	0.0352	0.0556		
Liaoning Manchu[31]	0.0139	0.0200	0.0254	0.0416	0.0075	0.0140	0.0188	0.0229	0.0057	0.0114	0.0092	0.0099	0.0063	0.0078	0.0056	0.0075	0.0143	0.0104	0.0079	0.0095	0.0197	0.0076	0.0098	0.0081	0.0123	0.0096	0.0105	0.0168	0.0125	0.0274	0.0265	0.0284	0.0338	0.0947	0.0306	0.0577	0.0381	0.0540	0.0074	

References:

[1] Wang M, Wang Z, He G, Jia Z, Liu J, Hou Y. (2018). Genetic characteristics and phylogenetic analysis of three Chinese ethnic groups using the Huaxia Platinum System. Sci Rep 8(1): 2429.
[2] Fan H, Wang X, Ren Z, He G, Long R, Liang A, Song T, Deng J. (2019). Population data of 19 autosomal STR loci in the Li population from Hainan Province in southernmost China. Int J Legal Med 133:429-431.
[3] Fan H, Du Z, Wang F, Wang X, Wen SQ, Wang L, Du P, Liu H, Cao S, Luo Z, et al. (2021). The forensic landscape and the population genetic analyses of Hainan Li based on massively parallel sequencing DNA profiling. Int J Legal Med 135:1295-1317.
[4] Yang L, Zhang X, Zhao L, Sun Y, Li J, Huang R, Hu L, Nie S. (2018) Population data of 23 autosomal STR loci in the Chinese Han population from Guangdong Province in southern China. Int J Legal Med 132(1):133-135.
[5] Liu QL, Chen YF, Huang XL, Liu KY, Zhao H, Lu DJ. (2017). Population data and mutation rates of 19 STR loci in seven provinces from China based on Goldeneye DNA ID System 20A. Int J Legal Med 131:653-656.
[6] Xie J, Shao C, Zhou Y, Zhu W, Xu H, Liu Z, Zhao Z. (2014). Genetic distribution on 20 STR loci from the Han population in Shanghai, China. Forensic Sci Int Genet 9:e30-31.
[7] Zou X, Li Y, Li P, Nie Q, Wang T, Hu Y, Zhu Y, Li J, Tang R. (2017) Genetic polymorphisms for 19 autosomal STR loci of Chongqing Han ethnicity and phylogenetic structure exploration among 28 Chinese populations. Int J Legal Med 131(6):1539-1542
[8] Xiao C, Zhang W, Wei T, Pan C, Huang D. (2016). Population data of 21 autosomal STR loci in Chinese Han population from Hubei province in Central China. Forensic Sci Int Genet 20:e13-e14.
[9] Xiang C, Huang L, Su S, Fan Y, Yu J, Huang Y, Xu B. (2016). Genetic variation of the 20 STRs in Han population from Yunnan-Kweichow plateau of China. Forensic Sci Int Genet 23:e10-e11.
[10] Zhang X, Liu L, Xie R, Wang G, Shi Y, Gu T, Hu L, Nie S. (2018). Population data and mutation rates of 20 autosomal STR loci in a Chinese Han population from Yunnan Province, Southwest China. Int J Legal Med 132(4):1083-1085.
[11] Wu L, Pei B, Ran P, Song X. (2017). Population genetic analysis of Xiamen Han population on 21 short tandem repeat loci. Leg Med (Tokyo) 26:41-44.
[12] Hu SP, Wu DQ, Xu XH, Liu JW, Li B. (2005). Genetic profile of 15 STR loci in the Min Nan population in Southeast China. Forensic Sci Int 152:263–265.
[13] Lian HH, Ge WD, Lin F, Li B. (2015). Genetic Polymorphisms of 21 Autosomal STR Loci of Fujian Han Population. Fa Yi Xue Za Zhi 31(3):211-214. Chinese.
[14] Sheng X, Wang Y, Zhang J, Chen L, Lin Y, Zhao Z, Li C, Zhang S. (2018). Forensic investigation of 23 autosomal STRs and application in Han and Mongolia ethnic groups. Forensic Sci Res 3(2):138-144.
[15] Wang HD, Wu D, Feng ZQ, Jing ZA, Li T, Guo QN, Zhang XP, Hou QF, Guo LJ, Kang B, et al. (2014). Genetic polymorphisms of 20 short tandem repeat loci from the Han population in Henan, China. Electrophoresis 35:1509-1514.
[16] Zhang Y, Li A, Zhao L, Liu J, Song J. (2011). Genetic polymorphisms of 20 Short Tandem Repeats of Hebei Han Population. Xing Shi Ji Shu. 02:48-50.
[17] Chen P, Wang B, He G, Song F, Luo H, Zhang L, Yu J, Han Y. (2017). Population genetic analyses of 20 autosomal STR loci in Chinese Han population from Tianjin in Northern China. Forensic Sci Int Genet 27:184-185.
[18] Zhang SS, Tang YQ, Zhang MX, Gao HM, Wang C, Wang YS, Han SY. (2018). Genetic Polymorphisms of 19 STR Loci in Populations of Three Culture Region in Shandong. Fa Yi Xue Za Zhi 34(3):264-269.
[19] Shen J, Kang H, Dong F, Guo J, Wang R. (2015). Research of genetic polymorphism on 19 STR loci in Han nationality in Shanxi province and forensic medicine application. Journal of Shanxi Police Academy 23: 63-66.
[20] Zhao W, Hu S, Wang T. (2015). Genetic polymorphisms of 19 STR loci of Pingliang Han of Gansu province. Chin J Forensic Med 30: 618–619.
[21] Zhang lili. (2017). Genetic profile of 21 STR loci in the Anhui Han in China. Zhongguo Fa Yi Xue Za Zhi 32(3):314-315. Chinese.
[22] Xing J, Xuan J, Wang B, Pang H, Li C, Ding M. (2011). Genetic polymorphism of 15 STR loci in a Manchu population in Northeast China. Forensic Sci Int Genet 5(3):e93-5.
[23] Hu S, Zhao W, Wang T. (2015). Genetic polymorphisms of 19 STR loci in Gansu Hui. Chin J Forensic Med 30: 620–621.
[24] Liu J, Wang Z, He G, Wang M, Hou Y. (2019). Genetic polymorphism and phylogenetic differentiation of the Huaxia Platinum System in three Chinese minority ethnicities. Sci Rep 9(1):3371
[25] Wang Z, Zhou D, Jia Z, Li L1, Wu W, Li C2, Hou Y. (2016). Developmental Validation of the Huaxia Platinum System and application in 3 main ethnic groups of China. Sci Rep 6:31075.
[26] Zhang X, Du L, Huang Z, Gu T, Hu L, Nie S. (2017). Genetic variation of 20 autosomal STR loci in three ethnic groups (Zhuang, Dai and Hani) in the Yunnan province of southwestern China. Forensic Sci Int Genet 31:e41-e42.
[27] Zhang X, Hu L, Du L, Nie A, Rao M, Pang JB, Xiran Z, Nie S. (2017). Genetic analysis of 20 autosomal STR loci in the Miao ethnic group from Yunnan Province, Southwest China. Forensic Sci Int Genet 28:e28-e29.
[28] Jin X, Wei Y, Chen J, Kong T, Mu Y, Guo Y, Dong Q, Xie T, Meng H, Zhang M, et al. (2017). Phylogenic analysis and forensic genetic characterization of Chinese Uyghur group via autosomal multi STR markers. Oncotarget 8:73837-73845.
[29] Zhang, L. et al. (2012). Genetic polymorphism of 19 STR loci in Xinjiang Barkol Kazakh population. Zhong Nan Da Xue Xue Bao Yi Xue Ban 37, 934–938.
[30] Zhao, L. et al. (2016). Genetic Polymorphisms of 19 STR Loci in Manchu population in Hebei Province. Chin. J. Forensic Med. 31: 72–73.
[31] Liu J, Guo L, Qi R, et al. (2013). Allele frequencies of 19 autosomal STR loci in Manchu population of China with phylogenetic structure among worldwide populations. Gene 529(2):282-287.
